# Conditioned Medium from Human Amnion-Derived Mesenchymal Stromal/Stem Cells Attenuating the Effects of Cold Ischemia-Reperfusion Injury in an In Vitro Model Using Human Alveolar Epithelial Cells

**DOI:** 10.3390/ijms22020510

**Published:** 2021-01-06

**Authors:** Vitale Miceli, Alessandro Bertani, Cinzia Maria Chinnici, Matteo Bulati, Mariangela Pampalone, Giandomenico Amico, Claudia Carcione, Eva Schmelzer, Jörg C. Gerlach, Pier Giulio Conaldi

**Affiliations:** 1 Research Department, IRCCS ISMETT (Istituto Mediterraneo per i Trapianti e Terapie ad Alta Specializzazione), 90127 Palermo, Italy; mbulati@ismett.edu (M.B.); pgconaldi@ismett.edu (P.G.C.); 2 Thoracic Surgery and Lung Transplantation Unit, Istituto Mediterraneo per i Trapianti e Terapie ad Alta Specializzazione, 90127 Palermo, Italy; abertani@ismett.edu; 3 Regenerative Medicine Unit, Fondazione Ri.MED, 90127 Palermo, Italy; cchinnici@fondazionerimed.com (C.M.C.); mpampalone@fondazionerimed.com (M.P.); gamico@fondazionerimed.com (G.A.); ccarcione@fondazionerimed.com (C.C.); 4 Department of Laboratory Medicine and Advanced Biotechnologies, IRCCS–ISMETT (Istituto Mediterraneo per i Trapianti e Terapie ad Alta Specializzazione), 90127 Palermo, Italy; 5 Department of Surgery, School of Medicine, McGowan Institute for Regenerative Medicine, University of Pittsburgh, Pittsburgh, PA 15219-3130, USA; schmelzere@upmc.edu (E.S.); joerg.gerlach@cellnet.org (J.C.G.); 6 Department of Bioengineering, School of Medicine, McGowan Institute for Regenerative Medicine, University of Pittsburgh, Pittsburgh, PA 15219-3130, USA

**Keywords:** lung ischemia-reperfusion injury, amnion-derived mesenchymal stem cells, ex vivo lung perfusion, conditioned medium

## Abstract

The clinical results of lung transplantation (LTx) are still less favorable than other solid organ transplants in both the early and long term. The fragility of the lungs limits the procurement rate and can favor the occurrence of ischemia-reperfusion injury (IRI). Ex vivo lung perfusion (EVLP) with Steen Solution^TM^ (SS) aims to address problems, and the implementation of EVLP to alleviate the activation of IRI-mediated processes has been achieved using mesenchymal stromal/stem cell (MSC)-based treatments. In this study, we investigated the paracrine effects of human amnion-derived MSCs (hAMSCs) in an in vitro model of lung IRI that includes cold ischemia and normothermic EVLP. We found that SS enriched by a hAMSC-conditioned medium (hAMSC-CM) preserved the viability and delayed the apoptosis of alveolar epithelial cells (A549) through the downregulation of inflammatory factors and the upregulation of antiapoptotic factors. These effects were more evident using the CM of 3D hAMSC cultures, which contained an increased amount of immunosuppressive and growth factors compared to both 2D cultures and encapsulated-hAMSCs. To conclude, we demonstrated an in vitro model of lung IRI and provided evidence that a hAMSC-CM attenuated IRI effects by improving the efficacy of EVLP, leading to strategies for a potential implementation of this technique.

## 1. Introduction

While lung transplantation (LTx) is the gold standard therapy for patients with end-stage lung disease, the long-term survival and outcomes after LTx are lower compared with those of other solid organ transplants [1]. LTx is still limited by both organ shortage and the fact that more than 80% of potential organ donors are not suitable for transplantation. Indeed, lungs are often discarded from donation because of organ damage such as neurogenic pulmonary edema, trauma, and infection, or in many cases (such as in donation after cardiac arrest) because the lungs cannot be fully evaluated before procurement [2].

Ischemia-reperfusion injury (IRI) can complicate LTx in more than 20% of the cases, contributing to significant morbidity and mortality [3,4]. IRI is driven by several inflammatory pathways (including release of reactive oxygen species, neutrophil activation, and production of proinflammatory factors) that are activated at the time of graft preservation and last until implantation. The activation of such pathways results in a significant damage of the alveolar capillary structures, which can lead to the development of early postoperative graft disfunction (PGD) and, ultimately, chronic lung allograft dysfunction (CLAD) [4,5]. In addition, hypothermic organ storage is also associated with oxidative stress and cell death in alveolar tissue, thus triggering the activation of proinflammatory pathways after lung transplantation [6]. Therefore, a reduction of IRI-induced side effects could significantly improve the success of LTx.

Recent works show that these clinical issues may be addressed by the use of normothermic ex vivo lung perfusion (EVLP) with Steen Solution (SS) [7,8], during which different treatments can also be used to reduce IRI [9,10]. SS is a perfusion solution with a suitable osmolarity, high dextran content, and antioxidative properties to ensure protection of the vascular endothelium from both macrophage and leukocyte activation as well as other injuries. During normothermic EVLP, the graft is warmed at the beginning of the procedure and then maintained at a temperature of 37 °C. Thus, unlike classic static cold storage (4 °C, which is the gold standard of graft preservation), EVLP keeps the lungs at physiological temperatures and allows for the evaluation of the lung function. EVLP aims to increase the number and, eventually, to improve the outcomes of LTx. In recent years, emerging evidence has shown that the results of EVLP can be improved with the use of pharmacological treatments to neutralize the activation of critical IRI-mediated processes [9,10,11]. Furthermore, the use of mesenchymal stromal/stem cell (MSC)-based therapies, which could include factor synthesis and regulation, also appears to be a very interesting approach to improve the EVLP technique.

Recent studies revealed that the use of MSCs has beneficial effects on lung epithelial cell viability [12] and mitigates both lung injury and inflammation in various experimental models [13,14]; different in vitro and in vivo results support the idea that MSCs and their derivatives have antibacterial and immunomodulatory properties that could decrease the onset of infection during lung transplant [15]. Indeed, different studies have shown that the infusion of MSCs in mice, rats, and pigs protects lung transplantation from IRI [16,17,18]. Furthermore, it has been revealed that MSC-based treatment during EVLP is associated with a decrease in adverse effects of both pig and human lung transplantation [19,20,21]. Nevertheless, in many of these studies, the infused MSCs were found in the lung parenchyma after intravascular administration, and this could present future constraints and concerns to the routine use of allogeneic MSCs during EVLP. Cell encapsulation without interaction with the recipient or cell-free therapies, including the use of a conditioned medium (CM), could be a new approach in which cells, unlike direct cell infusion methods, can help to reduce the degree of interaction between transplanted cells and the recipient, thereby maintaining MSC beneficial effects.

MSCs are found in several tissues, including bone marrow (BM) [22], adipose tissue [23], umbilical cord (UC) [24], and placenta [25], where these cells exhibit both immunomodulatory and angiogenic [26,27,28] as well as antioxidative properties [29]. In recent years, due to both the ethical issues regarding the source of MSCs and the fact that the sampling of some MSCs is very invasive, increasing evidence supports the use of neonatal tissues, such as UC tissue and placenta tissue (e.g., amniotic membrane), as interesting sources of MSCs [28,30,31]. This source has several advantages, including the number of cells that can be easily obtained without any invasiveness. Recently, Ertl et al. showed that different human placenta-derived MSCs (PD-MSCs), including human amnion-derived MSCs (hAMSCs), exert similar beneficial effects on wound closure and neovascularization in a mouse model, thus demonstrating the therapeutic potential of PD-MSCs in the field of regenerative medicine [32]. HAMSCs have been shown to have both immunosuppressive and angiogenetic abilities both in vitro and in vivo [33,34], and these are commonly ascribable to a paracrine mechanism mediated by the production of extracellular vesicles, such as exosomes and the secretion of cytokines, chemokines, and growth factors. In fact, several studies have indicated that hAMSCs secrete soluble factors with immunomodulatory and angiogenetic capacity [26,28,35,36]. Furthermore, it was shown that the in vivo administration of a CM derived from hAMSCs supports the repair process in mouse models of acute myocardial infarction [37], and it also reduced lung fibrosis in a bleomycin mouse model [38]. Moreover, prostaglandin-mediated immunosuppressive effects were found for a conditioned medium derived from hAMSCs [39].

Studies have shown that using MSC aggregation to form spheroids enhances their paracrine potential. Indeed, the enhanced secretion of anti-inflammatory cytokines by BM-MSC aggregates was thought to be responsible for the beneficial effects of MSCs in mice with myocardial infarcts [40]. Ylostalo et al. showed that MSCs in the spheroid form enhance their ability to produce prostaglandin E2, which in turn modulates macrophage responses [41]. Moreover, UC-MSCs and adipose-tissue-derived MSC aggregates showed a high secretion of angiogenic factors compared to monolayer MSCs [42,43]. Recently, we showed that hAMSCs grown as spheroids also potentiated their immunosuppressive and angiogenic properties compared to two-dimensional cultures [28]. Thus, these results identify aggregation of MSCs as a procedure to enhance their therapeutic potential.

**Figure 1:**
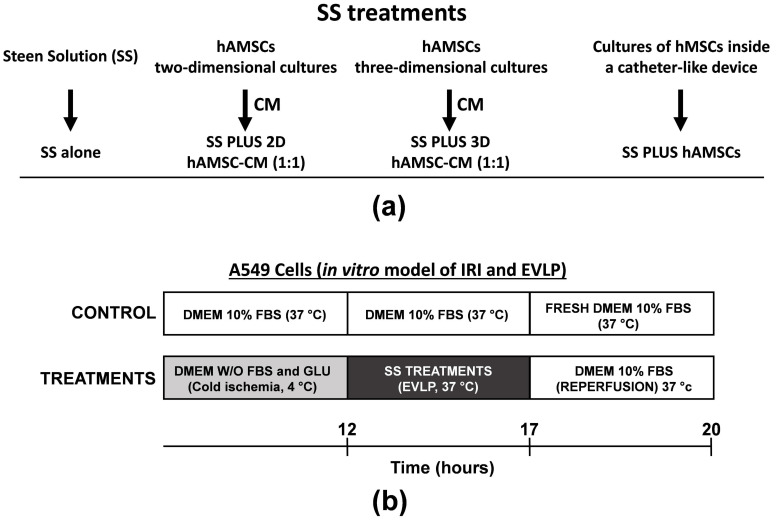
Treatments and experimental plan of in vitro model of ischemia-reperfusion injury (IRI) and ex vivo lung perfusion (EVLP). (**a**) Treatments tested in the study model. During in vitro EVLP, Steen Solution (SS) was used either alone or in combination with a conditioned medium (CM) derived from two-dimensional (2D) human amnion-derived mesenchymal stromal/stem cell (hAMSC) cultures (1:1 dilution ratio), three-dimensional (3D) hAMSC cultures (1:1 dilution ratio), or hAMSCs grown into a catheter-like device. (**b**) Experimental plane.

At present, approximately 1200 studies are registered at the US National Library of Medicine’s Clinical Trials database (clinicaltrials.gov), where MSCs are extensively tested in different areas of therapeutic applications, including organ transplantation. In our case, the use of MSCs aims at reducing IRI and acute inflammatory responses. In this study, as depicted in Figure 1, we investigate whether the hAMSCs encapsulated in a biocompatible and semipermeable membrane device (a temporarily implantable catheter-like device), or the CM produced by both 2D and 3D hAMSC cultures, administered as a treatment during in vitro EVLP, may prevent/mitigate the occurrence of IRI side effects in human alveolar epithelial cells. We hypothesize that the advantageous use of EVLP on LTx can be further enhanced by a treatment with hAMSC-conditioned medium during lung preservation/perfusion, leading to a decrease of IRI-mediated side effects.

## 2. Results

### 2.1. Characterization, Culture, and Differentiation of hAMSCs

**Figure 2:**
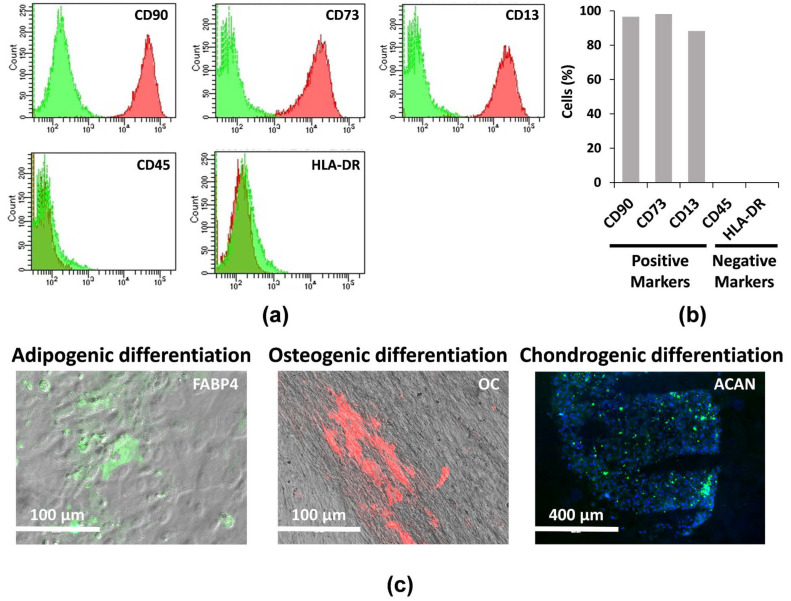
Phenotypic characterization and differentiation potential of hAMSCs. (**a**) Representative images and (**b**) quantification by flow cytometry analysis of both positive and negative surface marker in hAMSCs at passage 0. (**c**) Immunofluorescence staining of FABP4, OC, and ACAN in hAMSCs.

Here, hAMSCs derived from the amniotic membrane of placenta were analyzed by flow cytometry for both positive (CD90, 96.6%; CD73, 98.2%; CD13, 88.30%) and negative (CD45, 0.1%; HLA-DR, 0.1%) surface markers (Figure 2a,b). hAMSCs were then grown in two-dimensional (2D) cultures for 2 passages, and the multipotency of hAMSCs was verified by osteogenic, adipogenic, and chondrogenic differentiation (Figure 2c). Cells at passage 2 were grown in parallel in 2D cultures, inside a catheter-like device and in suspended state (three-dimensional, 3D) cultures where cells spontaneously aggregated and formed compact multicellular spheroids (Figure 1a).

### 2.2. Conditioned Medium Derived from 3D hAMSC Cultures Showing Increased Production of Both Growth and Immunomodulatory Factors

**Figure 3:**
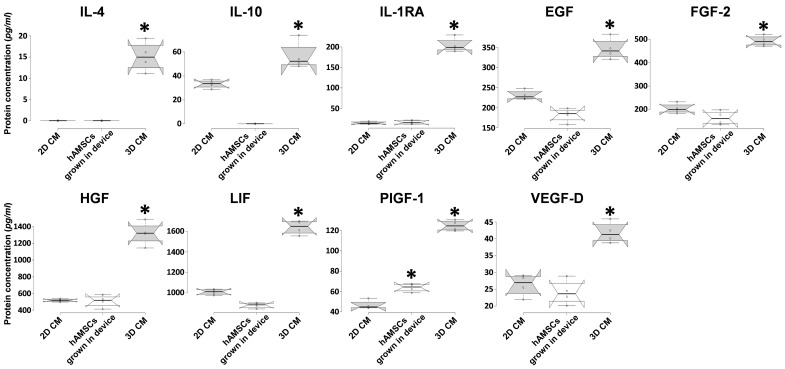
Secretion of cytokines and growth factors in two-dimensional (2D) hAMSC cultures, three-dimensional (3D) hAMSC cultures and hAMSCs grown in a catheter-like device. The conditioned medium (CM) was collected at 48 h for both the 2D and 3D CMs and at 8 h for hAMSCs grown in the device. The concentrations of cytokines and growth factors were determined by multiplex-microbead immunoassay. Box plots of four independent experiments are displayed, where the horizontal bar represents the median, the box represents the interquartile range (IQR), and the whiskers represent the maximum and minimum values. Comparisons made by Dunnet’s *t*-tests. * *p* < 0.05 compared with 2D CM.

To investigate whether the different culture systems alter the composition of CM, we used a multiplex-microbead immunoassay to analyze the secretion of different proteins in a CM produced by hAMSCs grown in both 2D and 3D cultures and in a catheter-like device. In particular, we evaluated the production of specific growth factors and cytokines, including IL-4, IL-10, IL-1RA, EGF, FGF-2, HGF, LIF, PIGF-1, and VEGF-D. As shown in Figure 3, compared with hAMSCs 2D cultures, 3D spheroids showed significantly enhanced secretory activity for IL-4, IL-10, IL-1RA, EGF, FGF-2, HGF, LIF, PIGF-1, and VEGF-D (15-, 1.6-, 14.5-, 1.5-, 2.5-, 2.5-, 1.6-, 2.7-, and 1.5-fold, respectively), while only PIGF-1 was significantly upregulated (1.4-fold) in hAMSCs grown in the device. It is worth noting that although hAMSCs were grown in the device for 8 h (while the CM from both 2D and 3D cultures were conditioned for 48 h), similar levels of IL-1RA, EGF, FGF-2, HGF, LIF, and VEGF-D were observed compared to 2D cultures (Figure 3).

### 2.3. Protective Effects of Encapsulated-hAMSCs and hAMSC-CM against Adverse Effects Induced by IRI on the Morphology and Viability of Human Alveolar Basal Epithelial Cells

**Figure 4:**
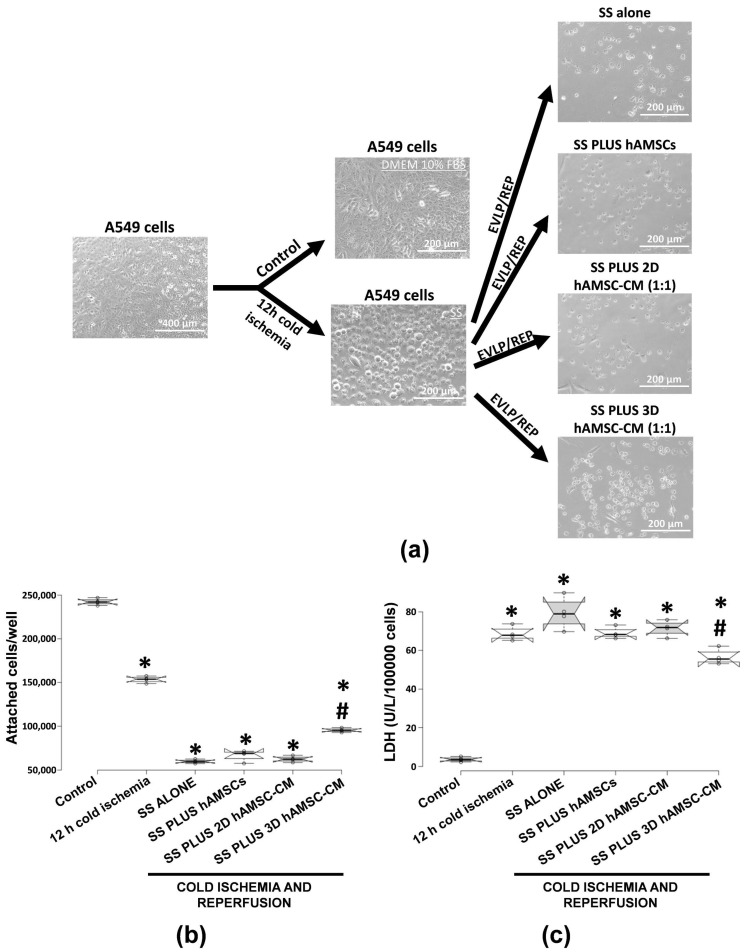
Effects of ischemia-reperfusion injury (IRI) on morphology, the number of attached cells, and lactate dehydrogenase (LDH) release in lung A549 cells. (**a**) Morphological changes of A549 cells observed under a light microscope after IRI. (**b**) Analysis of A549 cells attachment after IRI. (**c**) Analysis of lactate dehydrogenase (LDH) release in A549 cells after IRI. SS alone: A549 cells grown in Steen Solution alone during in vitro EVLP; SS PLUS hAMSCs: A549 cells grown in Steen Solution with encapsulated-hAMSCs during in vitro EVLP; SS PLUS 2D hAMSC-CM: A549 cells grown in Steen Solution diluted (1:1) with a CM derived from 2D hAMSC cultures during in vitro EVLP; SS PLUS 3D hAMSC-CM: A549 cells grown in Steen Solution diluted (1:1) with a CM derived from 3D hAMSC cultures during in vitro EVLP. Box plots of four independent experiments are displayed, where the horizontal bar represents the median, the box represents the interquartile range (IQR), and the whiskers represent the maximum and minimum values. Comparisons made by Dunnet’s *t*-tests. * *p* < 0.05 compared with the control. ^#^ *p* < 0.05 compared with SS alone treatment.

Human alveolar basal epithelial cells (A549 cells) were grown in parallel in both conventional cultures (DMEM 10% FBS at 37 °C) and cold ischemia (DMEM without FBS and glucose at 4 °C). According to the experimental plan detailed in Figure 1b, we tested the capabilities of encapsulated-hAMSCs and a CM derived from both 2D and 3D hAMSC cultures to alleviate the side effects of IRI on A549 cells. The treatment with 12-h cold ischemia was accompanied by characteristic morphologic changes, such as hypertrophic morphology (Figure 4a), and after 8 h of both in vitro EVLP and reperfusion, a drastic reduction in the number of attached cells was found (Figure 4a,b). In particular, after being washed, A549 cells displayed a 40% decrease in the number of attached cells after 12 h of cold ischemia compared to the control (Figure 4b). Then, the cells were preserved by in vitro EVLP with or without hAMSCs (encapsulated-cells or 2D/3D CM), and further enhancement of cell detachment was observed after 3 h of reperfusion. Cell attachment significantly decreased with SS treatment alone (75%), encapsulated-hAMSCs (72%), 2D hAMSC-CM (74%), and 3D hAMSC-CM (61%), compared with control (Figure 4b). Remarkably, only the treatment with 3D hAMSC-CM showed an increased percentage of attached cells compared with SS treatment alone (Figure 4b).

To investigate the potential protective effects of hAMSCs treatments, we also analyzed the lactate dehydrogenase (LDH) release of A549 cells after IRI. LDH is a stable cytoplasmic enzyme in all cells, and when the cell plasma membrane was damaged, LDH was rapidly released in the culture medium. Thus, the increase of LDH activity in culture medium can indicate the degree of cellular damage. After exposure to 12 h of cold ischemia, LDH activity increased in A549 cells to 66 U/L/1 × 10^5^ compared with the control (2.93 U/L/1 × 10^5^), indicating that the cells were markedly damaged. Interestingly, after in vitro EVLP and reperfusion, the cells released significantly more LDH in the SS alone group (85 U/L/1 × 10^5^), while the LDH levels remained stable in both SS plus hAMSCs (69 U/L/1 × 10^5^) and SS plus 2D hAMSC-CM (63 U/L/1 × 10^5^) compared with 12 h of cold ischemia treatment before EVLP/reperfusion. Interestingly, only the treatment with SS plus 3D hAMSC-CM induced a significant decrease of LDH to 54 U/L/1 × 10^5^ compared with the A549 cells after 12 h of cold ischemia (Figure 4c).

### 2.4. Effects of Encapsulated-hAMSCs and hAMSC-CM against Adverse Effects Induced by IRI on Apoptosis and Cell Cycle Progression of Human Alveolar Basal Epithelial Cells

**Figure 5:**
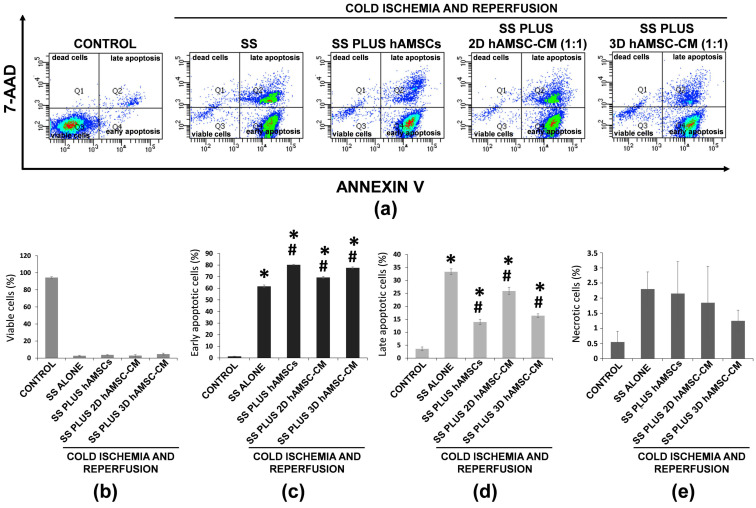
Effects of ischemia-reperfusion injury (IRI) on apoptosis/necrosis of A549 cells. (**a**) The levels of apoptosis and necrosis were determined by Annexin-V/7-AAD assays and analyzed by flow cytometry after IRI for each treatment. (**b**) The rates of viable cells. (**c**) Early apoptotic cells, (**d**) late apoptotic cells, and (**e**) necrotic cells were quantified. SS alone: A549 cells grown in Steen Solution alone during in vitro EVLP; SS PLUS hAMSCs: A549 cells grown in Steen Solution with encapsulated-hAMSCs during in vitro EVLP; SS PLUS 2D hAMSC-CM: A549 cells grown in diluted Steen Solution (1:1) with a CM derived from 2D hAMSC cultures during in vitro EVLP; SS PLUS 3D hAMSC-CM: A549 cells grown in diluted Steen Solution (1:1) with a CM derived from 3D hAMSC cultures during in vitro EVLP. All data are expressed as means ± SD. * *p* < 0.05 compared with control. ^#^ *p* < 0.05 compared with SS alone.

To examine whether encapsulated-hAMSCs or 2D/3D CM treatments have protective effects on IRI model of the epithelial lung, we analyzed apoptosis/necrosis using a double staining with annexin V and 7-ADD. Annexin V is used to detect early cell apoptosis, while 7-AAD permeates the cell membrane and dyes the cell nuclei red during the later stages of apoptosis in dead cells, thus differentiating apoptotic cells from necrotic cells. Figure 5 shows that the percentage of viable A549 cells exposed to ischemia/reperfusion insult, in both treated and untreated (SS alone) cells, decreased significantly from 95% to 2.7–4.7% (Figure 5b). Interestingly, different patterns of cell death were observed in relation to the various treatments. Indeed, a prevalent early apoptosis was observed in SS plus hAMSCs (encapsulated-hAMSCs) and SS plus 3D hAMSC-CM treatments (80% and 78%, respectively), while the levels of early apoptosis were, respectively, 62% and 70% in SS alone and SS plus 2D hAMSC-CM treatments (Figure 5c). Otherwise, the levels of late apoptosis were higher in SS alone and SS plus 2D hAMSC-CM treatments (33% and 26%, respectively) than in SS plus hAMSCs and SS plus 3D hAMSC-CM treatments (14% and 16%, respectively) (Figure 5d). Notably, all treatments with hAMSCs had higher levels of early apoposis and lower levels of late apoptosis compared with SS alone treatment (Figure 5c,d). Furthermore, a decreased percentage of necrotic cells (not significant) was observed in treatment with hAMSCs and 2D/3D CM compared with Steen Solution alone, with the lowest percentage seen in SS plus 3D hAMSC-CM treatments (1.25%) (Figure 5e).

**Figure 6:**
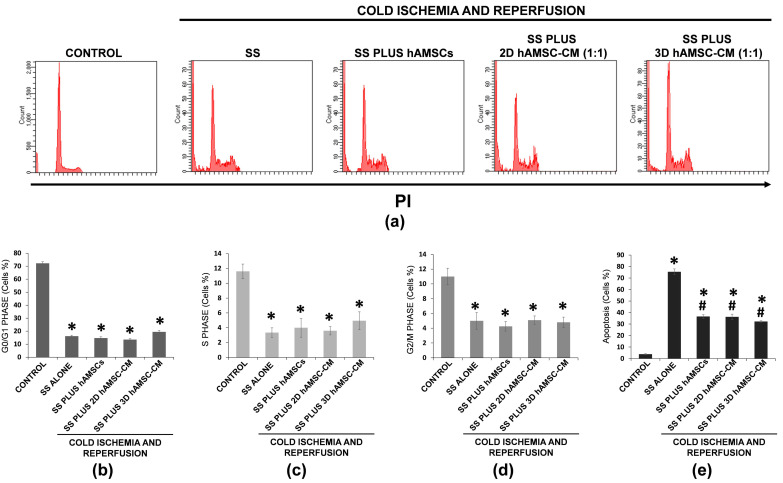
Effects of ischemia-reperfusion injury (IRI) on cell cycle of A549 cells. (**a**) Cycle arrest was determined by propidium iodide staining, and analyzed by flow cytometry after IRI for each treatment. The rates of cells in (**b**) G0/G1 phase, (**c**) S phase, (**d**) G2/M phase, and the rates of (**e**) apoptotic cells were quantified. SS alone: A549 cells grown in Steen Solution alone during in vitro EVLP; SS PLUS hAMSCs: A549 cells grown in Steen Solution with encapsulated-hAMSCs during in vitro EVLP; SS PLUS 2D hAMSC-CM: A549 cells grown in diluited Steen Solution (1:1) with a CM derived from 2D hAMSC cultures during in vitro EVLP; SS PLUS 3D hAMSC-CM: A549 cells grown in diluited Steen Solution (1:1) with a CM derived from 3D hAMSC cultures during in vitro EVLP. All data are expressed as means ± SD. * *p* < 0.05 compared with control. ^#^ *p* < 0.05 compared with SS alone.

We also evaluated the potential protective effects of hAMSCs and hAMSC-CM on cell cycle progression. We observed no significant differences in both S and G2/M phases between hAMSC/CM treatments and SS alone (Figure 6c,d) while, though not significant, an increased percentage of cells was observed in G0/G1 phase for Steen plus 3D hAMSC-CM treatments (19.5%) compared to SS alone (16.2%) (Figure 6b). Moreover, a significant decreased percentage of apoptotic cells was observed for hAMSC, 2D hAMSC-CM, and 3D hAMSC-CM treatments (37%, 36%, and 33%, respecitvely) compared with SS treatment alone (75%) (Figure 6e).

### 2.5. Effects of Encapsulated-hAMSCs and hAMSC-CM against Adverse Effects Induced by IRI on Expression of Inflammatory, Apoptotic, and Antioxidant Mediators in Human Alveolar Basal Epithelial Cells

We found that proinflammatory factors such as NF-kB, IL-1β, IL-6, IL-8, and CCL2 were significantly overexpressed in treatment with SS alone compared with the control (5.6-, 18.9-, 24.4-, 52.2-, and 33.7-fold, respectively), and all hAMSC/CM treatments were able to attenuate their upregulation. On the other hand, IL-4, the only anti-inflammatory factor analyzed, was significantly downregulated in treatment with SS alone compared with the control (5.9-fold), while its expression was not statistically different in all hAMSC/CM treatments.

**Figure 7:**
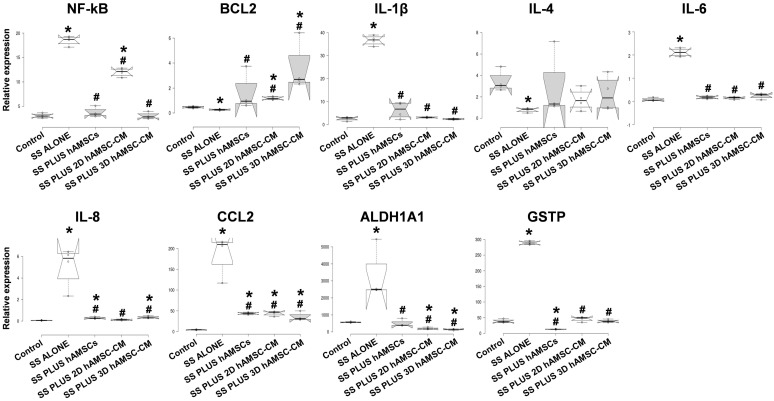
Expression analysis of immunomodulatory, apoptotic, and antioxidant genes in A549 cells. RT-PCR analysis was done for each treatment performed during in vitro ex vivo lung perfusion (EVLP) after ischemia-reperfusion injury (IRI). SS alone: A549 cells grown in Steen solution alone during in vitro EVLP; SS PLUS hAMSCs: A549 cells grown in Steen Solution with encapsulated-hAMSCs during in vitro EVLP; SS PLUS 2D hAMSC-CM: A549 cells grown in Steen Solution diluted (1:1) with a CM derived from 2D hAMSC cultures during in vitro EVLP; SS PLUS 3D hAMSC-CM: A549 cells grown in Steen Solution diluted (1:1) with a CM derived from 3D hAMSC cultures during in vitro EVLP. Transcript levels were normalized to those of GAPDH and expressed as fold change vs. untreated cells (control). Box plots of four independent experiments are displayed, where the horizontal bar represents the median, the box represents the interquartile range (IQR), and the whiskers represent the maximum and minimum values. Comparisons made by Dunnet’s *t*-tests. * *p* < 0.05 compared with the control. ^#^ *p* < 0.05 compared with SS alone treatment.

The antiapoptotic gene BCL2 was decreased in treatment with SS alone (1.8-fold) and overexpressed in encapsulated-hAMSCs, 2D hAMSC-CM, and 3D hAMSC-CM treatments compared with the control (2-, 2.32-, and 8.2-fold, respectively) (Figure 7). We observed that both ALDH1A1 and GSTP1 genes were upregulated in treatment with SS alone compared with the control (6.3- and 6.9-fold, respectively), while in all hAMSC/CM treatments, the expression levels for both genes were comparable to those observed in control (Figure 7).

## 3. Discussion

In this study, in order to improve EVLP performance, we developed a cell culture model that simulates the clinical processes of LTx, including cold lung ischemia and EVLP procedures; for this model, we tested the potential paracrine protective effects of hAMSCs even when these cells were grown either within a catheter-like membrane barrier device that enables communication with the culture medium and cells cultured in the dish but prevents a transfer of hAMSCs into the Petri-dish culture compartment, or in 3D cultures.

LTx improves the survival and quality of life in patients with end-stage lung disease, but despite the recent advances in both lung preservation techniques and operative management, lung transplant recipients still display the less favorable survival rates among solid organ recipients [44,45]. IRI significantly contributes to the development of acute lung injury (ALI), which leads to primary graft dysfunction (PGD) [46]. These processes are mediated, at least in part, by the induction of both inflammation and oxidative stress, which impair cellular homeostasis, leading to lung endothelial and epithelial cell death [47]. Moreover, the reperfusion of ischemic cells is often associated with the production of more reactive oxygen species, and the imbalance results in a subsequent inflammatory response [48]. Therefore, oxidative stress is concomitant with cellular injury, inflammation, and deregulated metabolism, which are involved in many IRI-mediated pathologies [29].

MSCs have been shown to exert both anti-inflammatory and antiapoptotic cytoprotective actions applicable in a variety of animal disease models, including lung diseases [49,50]. The mechanisms underlying these beneficial effects have been described in several studies. In particular, Lin et al. showed that adipose-derived MSCs protected rat kidneys from acute IRI by inhibition of both proinflammatory cytokines (TNF-α, NF-κB, IL-1β, MIF, PAI-1) and apoptotic protein (BAX, CASP3, PARP) [51]. Furthermore, in a rat model of hyperoxic lung injury, UC-derived MSCs were able to inhibit both inflammation and apoptosis [52]. In an in vitro model of simulated IRI (glucose-deprivation and hypoxia-re-oxygenation), BM-MSCs inhibited apoptosis by both the downregulation of proapoptotic protein (BAX and CASP3) and upregulation of antiapoptotic proteins, such as BCL2 [53]. Moreover, in a similar model, Song et al. showed that the human dental pulp-derived MSCs and its CM exerted cytoprotection against cell death of human astrocytes, and these effect were mediated, at least in part, by the increase of anti-inflammatory mediators, such as IL-10 [54]. Various reports have also shown that MSC-based treatments have significant protective effects against IRI in different phases of LTx procedures. In particular, it has been proven that MSCs are able to ameliorate IRI after cold ischemia in a mouse model of LTx [17,55]. Furthermore, the beneficial effects of MSCs have also been demonstrated when administered during EVLP procedures [21,56]. Nevertheless, in the field of LTx, unsolved issues—including malignant transformation, pulmonary embolism due to intravascular administration, and the fact that MSC properties depend on microenvironment signals [57,58]—have overshadowed the benefits of these MSC-based treatments. These difficulties could perhaps be overcome with the use of encapsulated and removable MSCs or by using cell-free therapy. Indeed, the paracrine effects of MSCs have been shown to be comparable to those of MSCs themselves [59,60], and various studies have demonstrated the paracrine beneficial effects of MSCs on different models of IRI [61,62]. In addition, we recently found that hAMSCs were capable of secreting both microvesicles and growth factors also when these cells were grown in a catheter-like device (encapsulated-hAMSCs) [35]. We also showed that hAMSCs grown as 3D spheroids potentiated the production of paracrine factors that are implicated in both immunoregulatory and cell growth pathways [28].

In our study, in an in vitro model of lung IRI, we examined the potential paracrine effects exerted by hAMSCs during a simulated EVLP, even when these cells were encapsulated in a membrane isolation device or grown as 3D spheroids. First, we analyzed the production of crucial factors involved in immunomodulation and cell growth/cytoprotection, and we observed that mainly 3D hAMSCs showed significantly enhanced secretory activity compared with 2D cultures. In particular, 3D hAMSCs overproduced IL-4, IL-10, IL-1RA, EGF, FGF-2, HGF, LIF, PIGF-1, and VEGF-D, compared to 2D cultures. Instead, encapsulated-hAMSCs only overproduced PIGF-1 significantly, while the expression of the other factors was comparable to that of 2D cultures. IL-1RA was shown to have protective effects through its anti-inflammatory and antioxidative mechanisms in an in vivo rat model of IRI [63], where IL-1RA not only effectively inhibited the expression of inflammatory factors, such as IL-1β, IL-6, and TNF-α, but it also decreased the death of damaged cells in intestinal tissues. It has been shown that both human and mouse pluripotent stem cells attenuate the apoptosis/necrosis and potentiate cell proliferation through different anti-inflammatory and growth factors, such as IL-10, FGF, EGF, and HGF [64,65]. Furthermore, Chen et al. showed that the activation of IL-4 signaling may serve as protection against IRI after stroke [66]. Interestingly, Li et al. reported that VEGF pathways activation is able to reduce oxidative stress and apoptosis [67]. Indeed, the treatment with PIGF-1 (aVEGFR-1 ligand) attenuates myocardial IRI by reducing both oxidative stress and apoptosis [68], while VEGF-D (a VEGFR-3 ligand) induces an antioxidant response of cells, which maintains the redox balance [69]. In addition, LIF has also been found to have protective effects against both oxidative stress and apoptosis [70]. Therefore, the release of these factors by MSCs has been associated with an enhanced resolution of IRI side effects, and our study supports this hypothesis. Our results indicate that the encapsulated-hAMSCs and, even more, the 3D hAMSC-CM, when administered during in vitro EVLP have protective effects against IRI induced in lung A549 cells. In our model, cold ischemia, the simulation of standard EVLP (SS alone), and reperfusion induced IRI effects on lung epithelial cells, characterized by both typical morphologic changes and an intense cell death, as shown in microscopic images, apoptosis/necrosis, cell attachment, and LDH assays (Figure 4 and Figure 5). Using mainly encapsulated-hAMSCs and 3D hAMSC-CM during in vitro EVLP as treatments for IRI attenuation, we found that we were able to increase the fraction of early apoptosis rather than late apoptosis, and principally a 3D hAMSC-CM treatment showed a significantly increased percentage of attached cells (61%) compared to SS treatment alone (75%) (Figure 4b). This suggests that cell attachment after ischemia and reperfusion can be affected by the treatment with hAMSC products administered during simulated EVLP. Furthermore, after IRI only the treatment with the 3D hAMSC-CM was able to significantly reduce the LDH levels compared to both cells before EVLP and cells after EVLP (Figure 4c). These data were corroborated by an analysis of genes involved in inflammatory, apoptotic, and detoxification pathways on injured lung cells. In particular, in A549 cells, IRI and standard EVLP (SS alone) produced a significant inflammatory response, as indicated by the upregulation of proinflammatory factors such as NF-kB, IL-1β, IL-6, IL-8, and MCP-1 (Figure 7), as shown in in vivo experimental models [71,72]. A549 cells treated with both encapsulated-hAMSCs and hAMSC-CM attenuated/inhibited the upregulation of these proinflammatory factors and, mainly 3D hAMSC-CM, induced a significant upregulation of the antiapoptotic gene BCL2, and a substantial inhibition of proinflammatory factor IL-1β compared with all the SS treatments (Figure 7). It is worth noting that various studies have highlighted the role of both BCL2 and IL-1β as crucial mediators of cytoprotection against cell death in several in vitro models of simulated IRI [53,54]. To understand the implications of altered gene expression patterns responsible for IRI, we also analyzed the expression of key genes involved in cytoprotection against oxidant stress, including ALDH1A1 and GSTP1. These enzymes are involved in detoxification of metabolites created during oxidative stress, such as those produced in ischemia reperfusion. In this regard, it has been shown that in a mouse model of diabetic neuropathy, BM-MSCs inhibited multiple parameters of neuroinflammation, preventing the production of proinflammatory cytokines, such as IL-1β and TNF-α, and increasing the secretion of anti-inflammatory cytokines such as IL-10 and TGF-β [73]. Moreover, this study found that neuroinflammation was attenuated by the reduction of oxidative stress signals, such as the transcription of nuclear factor erythroid 2-related factor 2 (NRF2), a crucial transcription factor that regulates key antioxidant genes, including ALDH1A1 and GSTP [74]. Interestingly, in an IRI model, it has been shown that antioxidant genes were upregulated in the reoxygenation phase protecting the cells against subsequent oxidative stress. In this model, a pretreatment with the antioxidant N-acetyl cysteine for 1 h prior to reoxygenation resulted in an inhibition of antioxidants’ upregulation [74].

Similarly, in our study, in addition to an overproduction of IL-10 and an inhibition of IL-1β by hAMSCs (mainly by 3D spheroids), we found that both ALDH1A1 and GSTP were upregulated in A549 cells after IRI in the treatment with SS alone, while their expression was comparable to that of control levels in the treatment with both encapsulated-hAMSCs and hAMSC-CM (Figure 7). These data also revealed potential protective effects of hAMSCs against oxidative stress that occurred during our IRI model.

Based on our observations, it is reasonable to suggest that during EVLP the use of a lung preservation solution enriched with paracrine components derived from hAMSCs might be considered a promising method of organ preservation, superior to the conventional use of SS alone. Furthermore, in our study, we also highlight other important aspects concerning MSC-based therapies. The first concerns the source of MSCs: Among MSCs, those extracted from placenta show numerous advantages, such as being abundant, easy to obtain without invasiveness from a medical waste, and readily cultured to a sufficient number for clinical use. Second, the feasibility of MSC encapsulation in a catheter-like device is a new therapeutic strategy for avoiding the interaction of MSCs with the recipient, and it also avoids ethical and biological questions concerning allografting. Third, the spontaneous cell aggregation appears to be an effective culture system for priming hAMSCs toward increased paracrine activity, which would further promote the therapeutic potential of these cells. The aim of improving EVLP techniques is to increase the number of suitable organs for LTx and to improve LTx outcomes. To achieve this aim, the use of encapsulated-cells or cell-free therapies offers a promising approach. Further in vitro and in vivo studies are needed to confirm these promising preliminary data and to explore the exact mechanisms by which hAMSCs exert their cytoprotective effects against IRI.

## 4. Materials and Methods

### 4.1. Cell Culture

Human type II alveolar epithelial cells (A549) were obtained from American Type Culture Collection (ATCC, Manassas, VA, USA). The cells were maintained in DMEM containing 25 mM glucose (Thermo Fisher Scientific, Waltham, MA, USA), supplemented with 10% fetal bovine serum (FBS), 2 mmol/L L-glutamine, 100 U/mL penicillin, and 100 μg/mL streptomycin (Thermo Fisher Scientific, Waltham, MA, USA) in a humidified atmosphere at 5% CO_2_, and 37 °C.

### 4.2. Isolation and Culture of Human Amnion-Derived Mesenchymal Stem/Stromal Cells

MSCs were isolated within 6 h of birth from the amnion of human term placenta of healthy donors. Written informed consent and details of the procedure were approved by ISMETT’s Institutional Research Review Board. Informed consent was obtained from each donor. To obtain the cells, the amnion was manually separated from the chorion and washed several times in PBS. After washing, it was cut into small pieces of 3 × 3 cm^2^, and each fragment was decontaminated in three different PBS solutions: (1) PBS supplemented with 2.5% Esojod (Esoform, Rovigo, Italy); (2) PBS supplemented with 500 U/mL penicillin, 500 mg/mL streptomycin, 12.5 mg/mL amphotericin B, and 1.87 mg/mL cefamezin (Pfizer, Milan, Italy); (3) PBS supplemented with 100 U/mL penicillin and 100 mg/mL streptomycin. Decontaminated fragments were incubated for 9 min at 37 °C in HBSS (Lonza, Basel, Switzerland) containing 2.5 U/mL dispase (Corning, New York, NY, USA) and then maintained for 5 min at room temperature in RPMI 1640 supplemented with 10% FBS (Thermo Fisher Scientific, Waltham, MA, USA). Thus, the amniotic fragments were digested with 0.94 mg/mL collagenase A (Roche, Mannheim, Germany) and 20 mg/mL DNase (Roche, Mannheim, Germany) for 2.5 h at 37 °C. Afterward, the digest was filtered with both 100 μm and 70 μm cell strainers (BD Falcon, San Jose, CA, USA), pelleted by centrifugation at 300 g for 10 min, and resuspended in RPMI 1640 medium supplemented with 10% FBS for cell counting. Harvested cells were cultured in polystyrene culture dishes (Corning, Corning, NY, USA) at 37 °C, 5% CO_2_ in Chang Medium (Irvine Scientific, Santa Ana, CA, USA). To obtain hAMSCs at different passages, the cells were plated at a density of 1 × 10^4^/cm^2^, and after reaching the confluence, adherent cells were trypsinized and then subcultured until passages 3–5. To study the potential paracrine effects of hAMSCs against IRI, the cells were grown in conventional cultures for the production of the 2D hAMSC-CM, or in suspension state for the production of the 3D hAMSC-CM, or in a catheter-like device consisting of a capillary fiber inserted in polystyrene culture dishes as described below [75].

### 4.3. Device Preparation

Hydrophilic MicroPES Type TF10 hollow capillary membrane fibers (Membrana, Germany) characterized by a membrane with an average pore diameter of 300 µm consisting of biocompatible polyethersulfone and polyvinylpyrrolidone were inserted into conventional polystyrene culture dishes (Falcon, Fisherbrand, Ottawa, ON, Canada) as previously shown [35]. The fibers allow for the transit of proteins with molecular weight up to MW 400,000. We attached the fibers to Petri dishes through embedding them on the outer end into Luer-Lock connectors and potting the hollow fibers with polyurethane so that they lead into the inside of the capillary, bridged to a hole in the side of the Petri dishes with silicone caoutchouc (Corning, Corning, NY, USA). To enable an injection of MSC into the lumen of the capillaries but preventing a transfer of MSC into the dishes, the inner ends of the capillaries were sealed with silicone caoutchouc.

### 4.4. Flow Cytometric Phenotypic Analysis

HAMSCs (10^6^) were detached using trypsin (Life Technologies, Carlsbad, CA, USA). Single cell suspensions were washed twice with a FACS buffer containing FBS and <0.1% NaN_3_ (BD Biosciences, San Jose, CA, USA, catalog No. 554656). Then, the cells were incubated on ice for 30 min with diverse fluorochrome-conjugated antibodies (dilution 1:20) against both positive markers (CD90-PE, CD73-APC, and CD13-APC) and negative markers (CD45-APC and HLA-DR-PE) (BD Biosciences, San Jose, CA, USA). Controls included nonstained cells and cells incubated with corresponding isotype controls (BD Biosciences, San Jose, CA, USA). Cells were then washed twice with the FACS buffer and analyzed using a FACSCanto II flow cytometer (Becton Dickinson, Franklin Lakes, NJ, USA) and FACSDiva 8.0.1 (Becton Dickinson, Franklin Lakes, NJ, USA) software. Cell debris and cell doublets were excluded by applying a forward versus side scatter gate.

### 4.5. Induction of Osteogenic, Adipogenic, and Chondrogenic Differentiation

Osteogenic differentiation and adipogenic differentiation were evaluated by growing hAMSCs for 14 days in αMEM with 10% FBS supplemented with osteogenic and adipogenic supplements, respectively (R&D Systems, Minneapolis, MN, USA). For chondrogenic differentiation, we used DMEM/F12 containing both ITS and chondrogenic supplement (R&D Systems, Minneapolis, MN, USA). We used a specific antibody panel consisting of anti-hFABP4 (adipocyte marker), anti-hOC (osteocyte marker), and anti-hACAN (chondrocyte marker) (R&D Systems, Minneapolis, MN, USA) to analyze the respective mature phenotypes by immunofluorescence. Samples were analyzed using an EVOS™ FL Digital Inverted Fluorescence Microscope (Thermo Fisher Scientific, Waltham, MA, USA).

### 4.6. Formation of Mesenchymal Stromal/Stem Cell Spheroids

Before the production of 3D-CM, hAMSCs at the second passage were cultured in 2 mL of DMEM serum-free medium at 5% CO_2_ and 37 °C (5 × 10^5^ cells/mL). The cells were maintained in a suspended state to allow for the formation of three-dimensional spheroids in a 6-well ultralow attachment plate (Corning, Corning, NY, USA) that facilitates spheroid formations and their maintenance.

### 4.7. Conditioned Media Preparation

For CM collection from 2D culture, the cells at the second passage were plated in a 100 × 17 mm dish (Nunc, Germany) at 5 × 10^5^ cells/mL in 10 mL of DMEM supplemented with 10% FBS until 90–95% confluence. The medium was then replaced with a serum-free DMEM medium (6 mL), and the cells were grown for 2 days. For CM collection from 3D cultures, we first observed the initial spheroid formation for 1 day; after this, the medium was changed, conditioned for 2 days, and finally collected. At the end of both cultures, each ml of collected medium was conditioned by 10^6^ cells. The supernatant from both culture systems was centrifuged and frozen at −80 °C until use.

### 4.8. Gene Expression Analysis

We performed real-time PCR using cDNA as the template in a 20 μL reaction mixture containing SYBR Select Master Mix (Thermo Fisher Scientific, Waltham, MA, USA) and a specific primer pair for the following genes: GAPDH, NF-kB, BCL2, IL-1β, IL-4, IL-6, IL-8, CCL2, ALDH1A1, and GSTP (Table 1). Briefly, total RNA was extracted with the RNeasy Mini Kit and treated with DNAse (QIAGEN, Hilden, Germany). Subsequently, 100 ng of RNA was transcribed with the high-capacity RNA-to-cDNAkit protocol (Thermo Fisher Scientific, Waltham, MA, USA) to produce single-stranded cDNA. Expression of mRNA was quantified by PCR using StepOnePlus Real-Time PCR System (Thermo Fisher Scientific, Waltham, MA, USA). GAPDH was used as a reference gene for the relative quantification, assessed by 2^−ΔΔCT^ calculation for each mRNA.

### 4.9. Protein Expression Analysis

The levels of different cytokine and growth factors in each conditioned medium (hAMSCs grown in both 2D and 3D cultures and in a catheter-like device) were determined using magnetic bead technology from Luminex™ with the ProcartaPlex Human Cytokine Chemokine Growth Factor (Affymetrix, Santa Clara, CA, USA) according to the manufacturer’s instructions. Concentration of each factor was calculated from standard curves.

### 4.10. In Vitro Protocol of Cold Ischemia, Normothermic Recovery (EVLP), and Reperfusion

In order to simulate the cold IRI shock, normothermic EVLP, and subsequent reperfusion, we replicated an in vitro protocol of cold starvation and normothermic recovery similar to that adopted in the clinical EVLP protocols for lung transplantation [76]. In particular, human alveolar epithelial A549 cells were maintained in DMEM without FBS and glucose at 4 °C for 12 h, followed by 4.5 h of recovery (EVLP), either in SS without or with encapsulated-hAMSCs, or in SS diluted (1:1) with 2D hAMSC-CM or 3D hAMSC-CM at 37 °C. A preventive preheating at room temperature was done before normothermic EVLP (30 min). Finally, a simulation of reperfusion was achieved by replacing SS with complete DMEM 10% FBS for 3 h at 37 °C (Figure 1b).

### 4.11. Analysis of Cell Death

First, we analyzed cell death by quantifying cell attachment with Countess II Automated Cell Counter (Thermo Fisher Scientific, Waltham, MA, USA). Briefly, the solution covering the cells was removed and the cell suspension was washed once with PBS for further analysis. Attached cells were detached (0.25% trypsin) at 37 °C, washed once with PBS, and an aliquot (10 µL) of cell suspension was quantified using the cell counter. Subsequently, we analyzed cell apoptosis/necrosis using Annexin-V and 7-AAD (BD Biosciences, San Jose, CA, USA) for cell staining. Their fluorescence was detected using a BD FACSCanto II instrument, and the data were analyzed with BD FACSDiva version 8.0.1 (BD Biosciences, San Jose, CA, USA). We also analyzed cell death by detection of the release of lactate dehydrogenase (LDH) enzymes in cell culture media. Enzyme activity was measured using Dimension Vista Intelligent Lab System (Siemens Healthcare Diagnostics, Norwood, MA, USA), according to the manufacturer’s instructions.

### 4.12. Cell Cycle Analysis

Cell cycle was measured with propidium iodide (PI) (Sigma, St. Louis, MO, USA) staining (50 µg/mL). In particular, hAMSCs were detached using trypsin (Life Technologies), washed once, and resuspended in 300 µL cold PBS. Then, the cells were fixed by adding 700 µL cold pure ethanol (−20 °C) dropwise during slow vortexing (70% final concentration). The cells were incubated on ice for 30 min. Fixed cells were washed once with PBS and subsequently resuspended in 1 mL PI (50 µg/mL) containing RNase (250 µg/mL) for 60 min, light-protected, at room temperature. PI fluorescence was detected using FACSCanto II flow cytometer (Becton Dickinson, Franklin Lakes, NJ, USA) and FACSDiva 8.0.1 (Becton Dickinson, Franklin Lakes, NJ, USA) software.

### 4.13. Statistics

All data were analyzed from at least triplicate in three independent experiments. Data from different groups were compared using computerized statistical software with the ANOVA test. When ANOVA revealed *p* < 0.05, the data were further analyzed with Dunnett’s *t*-test. Differences were considered statistically significant at *p* < 0.05.

## 5. Conclusions

In a setting of in vitro IRI, we found that human lung alveolar cells were significantly impaired by both cold ischemia and reperfusion. Our data showed that specific factors secreted by hAMSCs administered before reperfusion can have protective effects on ischemic-injured pulmonary cells by delaying cells entering apoptosis and preserving cell viability and membrane integrity, partially through the downregulation of inflammatory factors and the upregulation of antiapoptotic factors. In particular, 3D hAMSC cultures showed increased production of both immunosuppressive (IL-4, IL-10, IL-1RA, HGF, and LIF) and growth factors (EGF, FGF-2, HGF, and PIGF-1), inhibiting crucial inflammatory factors (NF-kB, IL-1β, IL-6, IL-8, MCP-1) and inducing a key antiapoptotic factor (BCL2) in injured cells. To increase the organs suitable for LTx and to improve the LTx outcome, there is the need to improve the EVLP techniques. In this study, we showed that the addition of the hAMSC-CM to the conventional lung preservation solution (SS) could perhaps lead to an improvement in EVLP efficacy, and our in vitro model of lung IRI might be useful in establishing a suitable treatment setting in in vivo EVLP.

## Figures and Tables

**Table 1:** Primer sequences for real-time PCR analysis.

Gene	Forward (5′-3′)	Reverse (5′-3′)	GenBank Accession ID	Amplicon Length (bp)
NF-kB	TACTCTGGCGCAGAAATTAGGTC	CTGTCTCGGAGCTCGTCTATTTG	NM_003998.3	263
BCL2	ATCGCCCTGTGGATGACTGAG	CAGCCAGGAGAAATCAAACAGAGG	NM_000633.2	128
IL-1β	TCCAGGGACAGGATATGGAG	TCTTTCAACACGCAGGACAG	NM_000576.2	132
IL-4	CTTTGCTGCCTCCAAGAACAC	GCGAGTGTCCTTCTCATGGT	NM_000589.3	96
IL-6	TGTGAAAGCAGCAAAGAGGC	TGGGTCAGGGGTGGTTATT	NM_000600.4	275
IL-8	CCACCGGAGCACTCCATAAG	GATGGTTCCTTCCGGTGGTT	NM_000584.3	96
CCL2	CGCGAGCTATAGAAGAATCAC	TTGGGTTGTGGAGTGAGTGT	NM_002982.3	176
ALDH1A1	CAAGATCCAGGGCCGTACAA	GGAGGAAACCCTGCCTCTTTT	NM_000689.4	233
GSTP	CCCTACACCGTGGTCTATTTCC	GAGGCTTTGAGTGAGCCCT	NM_000852.3	133
GAPDH	TCAAGAAGGTGGTGAAGCAGG	ACCAGGAAATGAGCTTGACAAA	NM_002046.6	167

## Data Availability

The datasets used and analyzed are available from the corresponding author on reasonable request.

## Abbreviations

LTx	Lung transplantation
IRI	Ischemia-reperfusion injury
EVLP	Ex vivo lung perfusion
MSCs	Mesenchymal stromal/stem cells
hAMSCs	Human amnion-derived mesenchymal stromal/stem cells
CLAD	Chronic lung allograft dysfunction
PGD	Primary lung graft dysfunction
ALI	Acute lung injury
CM	Conditioned medium
PD-MSCs	Placenta-derived mesenchymal stromal/stem cells
2D	Two-dimensional
3D	Three-dimensional
LDH	Lactate dehydrogenase
PI	Propidium iodide
NRF2	Nuclear factor erythroid 2-related factor 2
7AAD	7-Aminoactinomycin D
